# The historical impact of anthropogenic air-borne sulphur on the Pleistocene rock art of Sulawesi

**DOI:** 10.1038/s41598-022-25810-1

**Published:** 2022-12-13

**Authors:** Michael K. Gagan, Halmar Halide, Raden Cecep Eka Permana, Rustan Lebe, Gavin B. Dunbar, Alena K. Kimbrough, Heather Scott-Gagan, Dan Zwartz, Wahyoe S. Hantoro

**Affiliations:** 1 School of Earth, Atmospheric and Life Sciences, University of Wollongong, Wollongong, NSW 2522 Australia; 2 School of Earth and Environmental Sciences, The University of Queensland, St Lucia, QLD 4072 Australia; 3 Australasian Earth Systems, P.O. Box 1290, Canberra, ACT 2601 Australia; 4 Departemen Geofisika, Fakultas Matematika Dan Ilmu Pengetahuan Alam, Universitas Hasanuddin, Makassar, 90245 Indonesia; 5 Departemen Arkeologi, Fakultas Ilmu Pengetahuan Budaya, Universitas Indonesia, Depok, 16424 Indonesia; 6 Balai Pelestarian Cagar Budaya Sulawesi Selatan, Makassar, 90111 Indonesia; 7 Antarctic Research Centre, Victoria University of Wellington, Wellington, 6140 New Zealand; 8 Centre for Science in Society, Victoria University of Wellington, Wellington, 6140 New Zealand; 9 Research Center for Geotechnology, Indonesian Institute of Sciences, Bandung, 40135 Indonesia

**Keywords:** Environmental impact, Archaeology, Climate change

## Abstract

The Maros-Pangkep karst in southwest Sulawesi, Indonesia, contains some of the world’s oldest rock art. However, the Pleistocene images survive only as weathered patches of pigment on exfoliated limestone surfaces. Salt efflorescence underneath the case-hardened limestone substrate causes spall-flaking, and it has been proposed that the loss of artwork has accelerated over recent decades. Here, we utilise historical photographs and superposition constraints to show that the bulk of the damage was present before 1950 CE, and describe the role of anthropogenic sulphur emissions in promoting gypsum-salt efflorescence and rock art decay. The rock art shelters have been exposed to domestic fire-use and intensive rice cultivation with post-harvest burning of straw for hundreds (if not thousands) of years, both of which release chemically reactive sulphur oxides for gypsum formation, with cumulative effects. Analysis of time-lapse photography indicates that the rate of rock art loss may be on the decline, consistent with the history of fire-use in southwest Sulawesi. At present, vandalism and sulphur emissions from diesel-powered traffic and cement-based infrastructure development constitute localised threats. Our findings indicate that there are grounds for being cautiously optimistic that targeted conservation measures will ensure the longevity of some of our oldest artistic treasures.

## Introduction

**Figure 1:**
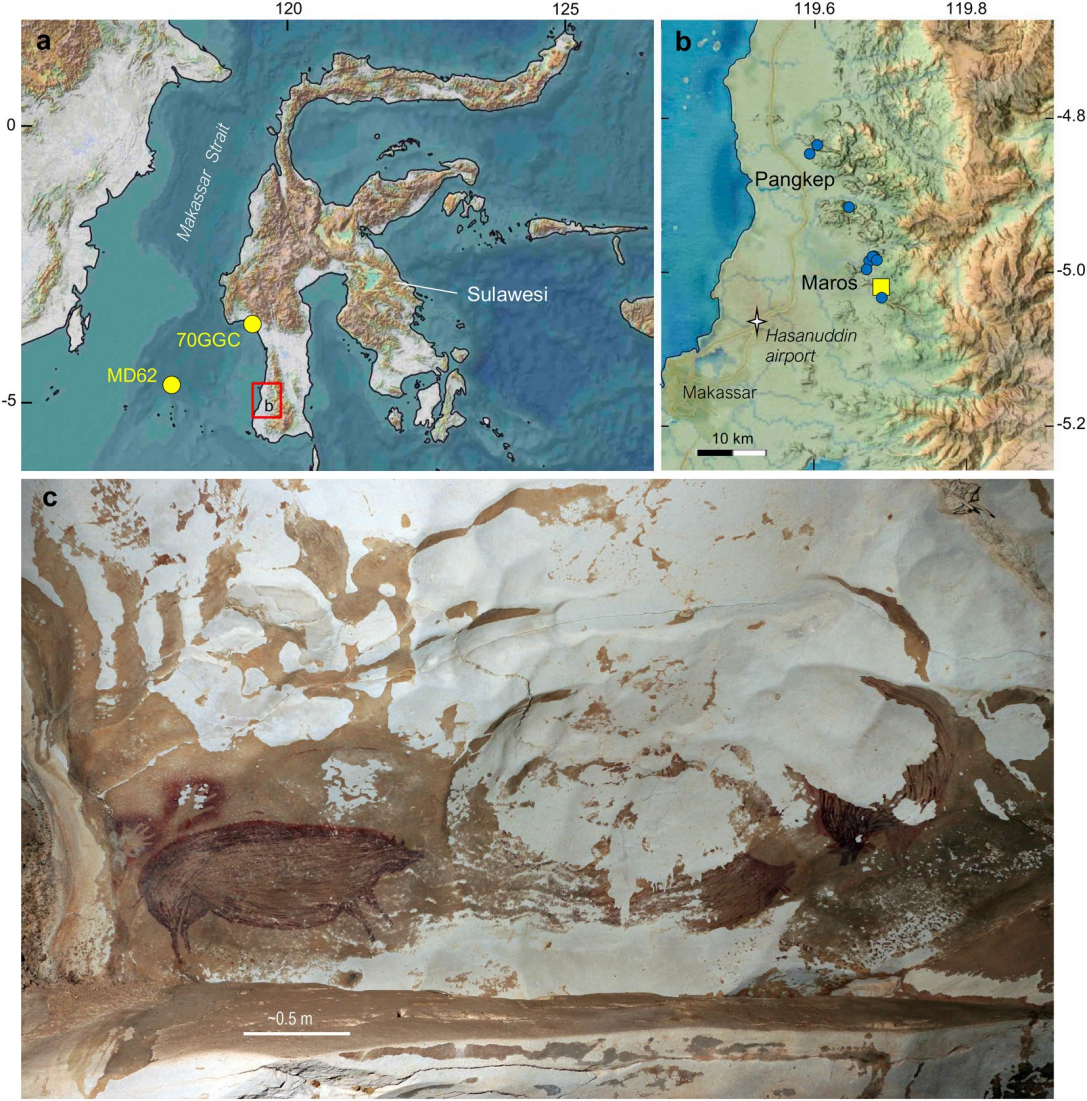
Physiographic attributes of the study area. (**a**) Location of the study area (red rectangle) on the southwestern peninsula of Sulawesi. Yellow circles show marine sediment cores used for sea-surface temperature reconstructions discussed in this study. (**b**) Physiography of the Maros-Pangkep karst and locations of rock art sites specific to this study (blue circles). Yellow square shows Gempa Bumi Cave with a speleothem δ^18^O reconstruction of local rainfall. (**c**) Exfoliation of cave surfaces (pale areas) and damage to Pleistocene paintings of pigs in the Leang Tedongnge rock shelter. Base maps were created by A. K. Kimbrough in QGIS 3.20 (https://qgis.org/en/site/) and Adobe Illustrator CC using Shuttle Radar Topography Mission 1 Arc-Second Global by NASA/NGS/USGS (2015-01-01 EPSG4326_31m). Photo credit: Auto-adjusted photograph by Basran Burhan (Griffith University) available at: https://commons.m.wikimedia.org/wiki/File:Leang_Tedongnge_rock_art_panel_credit_Basran_Burhan.jpg#.

The limestone terrane of the Maros-Pangkep karst on the Indonesian island of Sulawesi contains representational rock art dating to a minimum age of 45.5 thousand years ago (ka)^1,2^ (Fig. 1). More than 240 caves and rock shelters harbouring an irreplaceable archive of imagery have been documented^3^. The exquisite older rock art is characterised by red/mulberry hued pigments and includes hand stencils, figurative depictions of animals, and human/animal composites^1,2^. However, the degraded state of some of the Pleistocene hand stencils and figurative art was already conspicuous during an archaeological survey in 1950^4^. And in the mid-1980s, restoration of heavily damaged artwork was undertaken in the Leang Petta Kere and Leang Sumpang Bita rock shelters^5,6^. Since then, the Indonesian archaeology community has produced a body of evidence on the damage and described a range of potential physical, chemical and biological causes^3,7–14^.

A recent study demonstrated that salt efflorescence is the underlying cause of exfoliation of the case-hardened limestone surfaces that form the substrate for the older Pleistocene paintings^15^. Case-hardening is produced by the precipitation of solutes at the limestone’s surface to form a durable crust. However, geological salts, notably the sulphate mineral gypsum (CaSO_4_.2H_2_O), can easily accumulate in the void spaces created below the case-hardened surface by the dissolution of cement matrices^15^. Changes in temperature and relative humidity cause the gypsum crystals to expand and contract and, over time, spall-flakes are dislodged from the limestone surface. As a result, much of the older Maros-Pangkep artwork is patchy and poorly preserved (Fig. 1c).

Huntley et al.^15^ proposed that the Pleistocene rock art has weathered at an alarming rate in recent decades. However, systematic assessments of the rate of rock art loss in selected cave sites have only been underway since 2018^3^. Therefore, as yet, there are no definitive measurement results to validate an acceleration of panel loss. We raise the point because it has been proposed that increases in the frequency and severity of El Niño-induced droughts in Sulawesi pose the greatest threat to the Maros-Pangkep rock art^15^. Clearly an evaluation of the current rate of rock art loss within the broader context of climate variability is crucial for understanding how to protect the rock art.

Here, we evaluate the history of rock art loss within the context of climate change in southwest Sulawesi over the last 40 kyr. The available palaeoclimate records and instrumental records of the El Niño-Southern Oscillation (ENSO) do not support climate change on its own as the driver of gypsum efflorescence. Instead, our analysis of historical photographs of rock art panels shows that most of the limestone exfoliation has been inactive since 1950 CE. Without a clear connection to climate change, the degradation of the Maros-Pangkep rock art requires more proximal causes. The triggering of gypsum crystal growth and weathering of carbonate building stone by atmospheric SO_2_ pollution in urban settings is well documented across a broad range of climatic settings^16–20^. We present the case for anthropogenic sulphur emissions from prehistoric in-cave use of fire and agricultural burning and, more recently, combustion of diesel fuels and cement-based infrastructure development, as the underlying drivers of gypsum efflorescence and exfoliation of the Maros-Pangkep art.

## Results and discussion

### Climate change over the last 40,000 years

**Figure 2:**
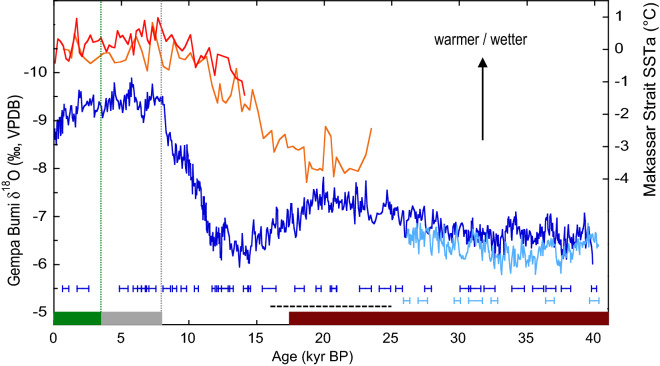
Climate change and human presence in southwest Sulawesi over the last 40 kyr. Orange curves show sea-surface temperature anomalies (SSTa relative to the last 2 kyr) based on Mg/Ca in planktonic foraminifera in sediment cores from Makassar Strait^21,22^. Blue curves show stalagmite δ^18^O records for Gempa Bumi Cave as an indicator of austral summer monsoon rainfall (corrected for changes in seawater δ^18^O)^24^. U-Th dates (in kyr BP) with 2σ errors are shown below the δ^18^O curves. The Pleistocene age-ranges for U-Th dated rock art^1,2^ and the earliest modern human skeletal remains^45^ at Maros-Pangkep are indicated by the mulberry bar and dashed line, respectively. Toalean hunter-gatherers occupied the area from ~ 8–2 ka^65^ (grey bar) and overlapped Austronesian farmers starting ~ 3.5 ka^35^ (green bar).

The available marine and terrestrial palaeoclimate records for southwest Sulawesi provide histories of local sea-surface temperature (SST) and summer monsoon rainfall over the last 40 kyr (Fig. 2, see Methods). Analysis of Mg/Ca in planktonic foraminifera in marine sediment cores from Makassar Strait shows that SSTs (and thus local surface air temperatures) were ~3-4 °C cooler between 24 and 17 ka^21–23^. SSTs increased during the deglaciation and after ~10 ka they were generally equal to or up to 0.5 °C warmer than pre-industrial times.

Oxygen-isotope ratios (δ^18^O) in speleothems from the Maros karst show a substantially drier austral summer monsoon between ~40 and 12 ka (Fig. 2), consistent with the relatively cool local SSTs and exposure of the Sunda-Sahul shelves during glacial times^24^. Pervasive dry conditions from ~33 to 16 ka are also a feature of proxies of surface runoff and vegetation at Lake Towuti ~150 km northeast of Maros^25^. The Maros speleothem δ^18^O record indicates that summer monsoon rainfall strengthened rapidly after ~12 ka in response to the inundation of Sunda-Sahul and reached (or exceeded) modern values by ~8 ka.

In sum, the available palaeoclimate records show that recent changes in SST and austral summer monsoon rainfall are well within the range of climate change endured by the Pleistocene rock art. The range of ENSO variability over the last 40 kyr is not as well documented. Proxy records of ENSO activity during the Last Glacial Maximum and early Holocene are generally not in good agreement^26,27^. However, there is reasonably good agreement between palaeoclimate records and models that ENSO was periodically weaker than at present from ~6 to 3 ka^26,28^. Higher-resolution palaeoclimate records for the last millennium indicate that ENSO variability was modulated on centennial to multi-decadal scales^27,28^.

### Recent ENSO variability

**Figure 3:**
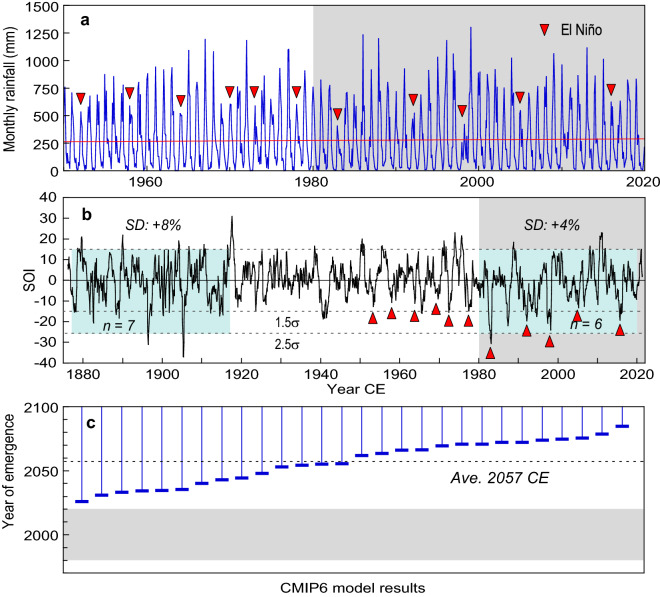
Rainfall in the study area and ENSO variability. (**a**) Monthly rainfall in the Maros area for 1950–2019^63^ showing no significant trend toward the present. Red triangles indicate reduced rainfall during moderate-strong El Niño years. (**b**) The Southern Oscillation Index (SOI) for 1876–2021 (3-month running mean). The two 40-year epochs of strong SOI variability (1877–1916 and 1980–2019, blue) with 6–7 El Niño years have essentially the same standard deviation (4–8% higher than average). (**c)** Time of emergence for anthropogenic signals in ENSO variability over the Niño 3.4 region in 28 Coupled Model Intercomparison Project Phase 6 (CMIP6) model simulations^26^. Stronger rainfall variability is predicted to emerge in the mid-late twenty-first century when the signal-to-noise ratio exceeds 1.5 under the highest greenhouse gas emission scenario (SSP5-8.5). Grey shading shows the post-1980 period when rock art degradation is thought to have accelerated^15^.

Sulawesi is susceptible to dry spells caused by El Niño events^29^. Huntley et al.^15^ proposed that a recent increase in El Niño-induced droughts due to anthropogenic climate change promoted the salt efflorescence that damages the Pleistocene rock art. However, the available palaeo-ENSO and instrumental climate records do not show a sustained trend to higher ENSO variability that can be distinguished from multi-decadal variability^27^. To illustrate this, we analysed monthly average rainfall in southwest Sulawesi and the Southern Oscillation Index (SOI). Quality-controlled instrumental rain-gauge station data for southwest Sulawesi extend back to 1950, but the SOI covers 145 years from 1876 to 2021 (Fig. 3, Supplementary Fig. 1, Methods). The SOI provides a consistent record of large-scale surface air pressure gradients through time whereas SST observations of El Niño in the tropical Pacific are less reliable before 1950^30^.

Figure 3 shows that dry spells in southwest Sulawesi are well correlated with positive SOI intervals (El Niño events) since 1950, consistent with previous findings^31^. Given the strength of the correlation, the SOI can be used as an indicator of rainfall variability in Maros-Pangkep back to 1876. The key point here is that there is no sustained increase in SOI amplitude or El Niño event frequency toward the present. The two 40-year epochs of strongest SOI variability separated by ~100 years (1877-1916, 1980-2019), with 6-7 El Niño years each, are essentially the same, consistent with the findings of other studies^27^.

The fact that ENSO variability since 2000 CE has been generally weaker than in the 1980s and 1990s raises questions about when projected changes in ENSO might emerge above the background level of climate variability. Most of the current generation of climate models indicate that significant strengthening of ENSO rainfall variability will emerge in the mid-late 21st century^26^ (Fig. 3). However, recent high-resolution modelling with a more realistic tropical Pacific mean state suggests that ENSO could weaken under a quadrupling of atmospheric CO_2_^32^, or even become more La Niña-like^33^. Also, new millennial-length model simulations that extend beyond centennial-scale internal climate variability suggest that CO_2_ forcing decreases ENSO amplitude over the long term^34^.

In summary, the available climate records show that the Maros-Pangkep rock art has endured a large range of climate change over the last 40 kyr that exceeds the temperature, rainfall and ENSO variability in recent decades. In particular, El Niño event amplitude and frequency were not unusual during recent decades when the degradation of the Maros-Pangkep rock art is thought to have accelerated.

### Neolithic arrival of rice agriculture and rock art degradation

**Figure 4:**
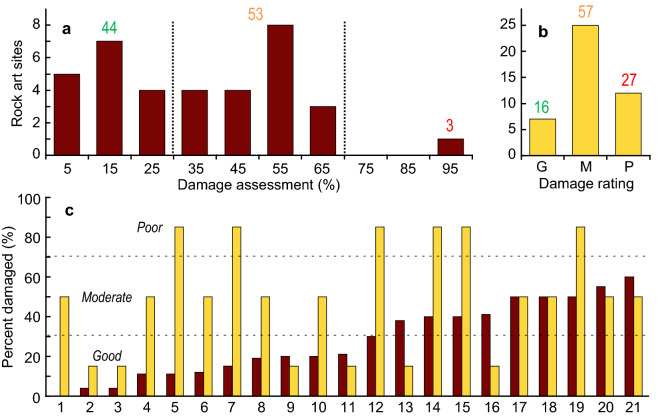
Assessments of rock art degradation in Maros-Pangkep. (**a**) Summary of damage assessments in 2003–2005 for 727 hand stencils in 36 cave sites^7^. (**b**) Damage ratings assigned to 44 sites in 2013 using broader criteria^11^ (see Methods). Coloured numbers indicate the percentage of sites in each damage category. (**c**) Comparison of the two sets of assessments for 21 rock art sites in common (numbers identify sites noted in Supplementary Table 1). The damage ratings in (**b**) have been assigned broad percentage ratings: “good” (< 30% damaged), “moderate” (30–70% damaged), “poor” (> 70% damaged). Both datasets show that the degree of rock art degradation is highly variable among sites.

Knowledge of the long-term history of the Maros-Pangkep rock art loss is essential to put the observed damage into context and evaluate potential causes. A recent conservation study of 340 rock art images in five sample caves showed that 93% are damaged^3^. Twelve types of physical, chemical and biological causes have been identified, with exfoliation (89%) and algal growth (31%) dominant^3^. Importantly, the available assessment data on rock art degradation in Maros-Pangkep, based on broad criteria, show significant differences in the preservation of artwork among shelters^7,11^ (Fig. 4, see Methods). This finding is consistent with local environmental controls, rather than climate change, as the primary cause of the degradation of the rock art.

The age-range of the limestone exfoliation, and how much of it is currently active, are unknown. However, Aubert et al.^1^ noted the potential antiquity of the exfoliation. They surmised that a relatively recent Austronesian style of art painted on exfoliated surfaces among the residual Pleistocene art marked the arrival of farming communities and rice cultivation in Sulawesi ~3,500 years ago^35^. The Austronesian art is generally painted with black charcoal-based pigment. Recent AMS radiocarbon dating of a drawing of a human figure in the Austronesian style yielded an age of 1583‒1428 calBP^15^. This is a maximum age for the artwork, but the date is consistent with the artistic style, and indicates that limestone exfoliation was underway in the Maros-Pangkep rock art shelters by ~ 1500 years ago.

**Figure 5:**
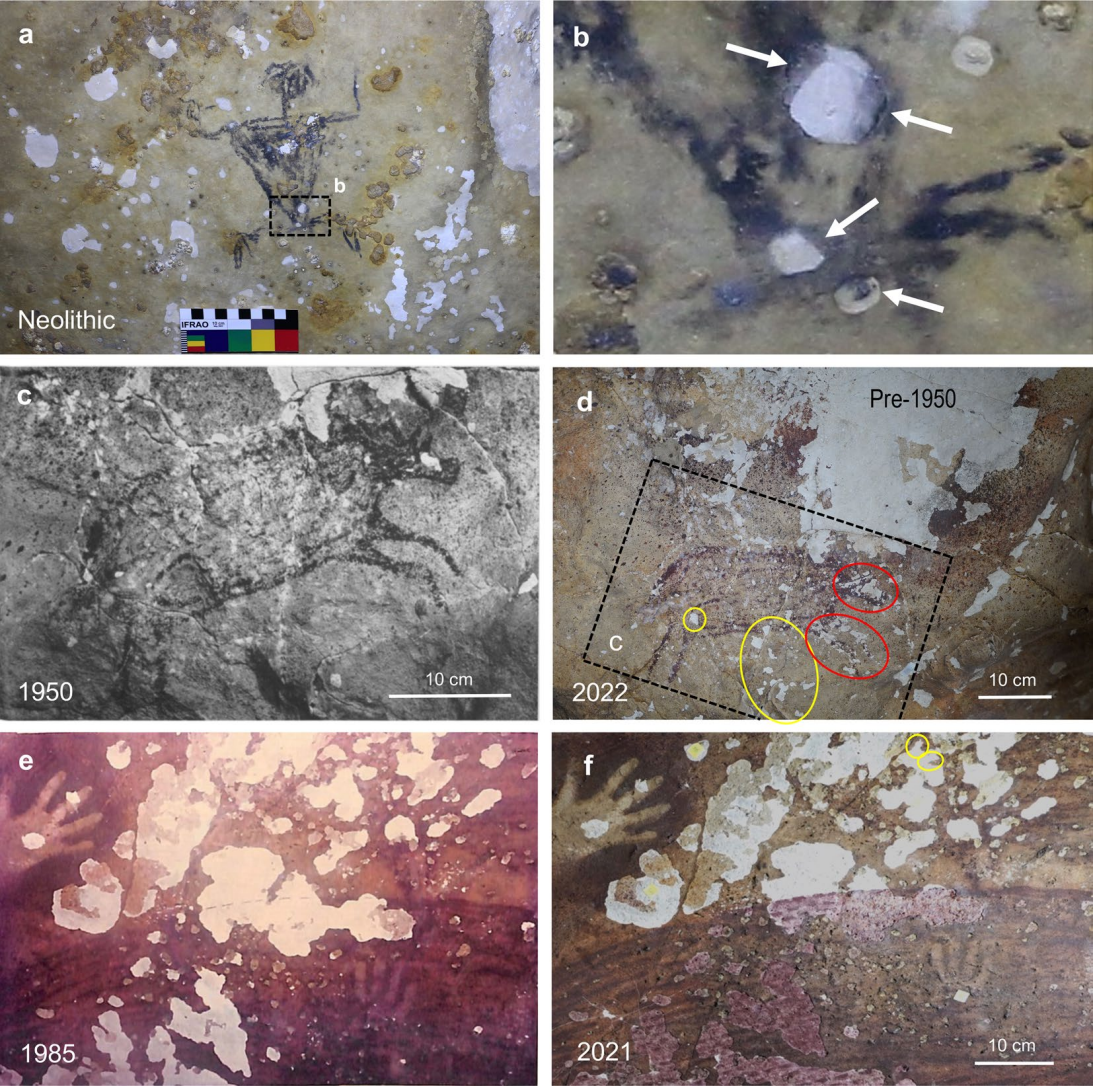
Historical photographs of rock art exfoliation in the Maros karst. (**a**,**b**) Austronesian-style anthropomorphic figure with charcoal-based pigment overlying ancient spall-scars in Leang Sampeang. (**c**,**d**) Surface exfoliation around a painting (the “leaping boar”) in Leang Pettae in 1950 (ref.^53^, Plate 31) and on 31 March 2022. Yellow ovals indicate exfoliation since 1950; red ovals show possible vandalism (see text). (**e**,**f**) Condition of a painting of a pig in Leang Petta Kere in October 1985 before restoration work (ref.^5^, Photo 13) and on 9 September 2021. The similarity of exfoliation across treated and untreated areas shows that very little has changed over 36 years. Photo credits: (**a**,**b**,**d**) R. Lebe; (**c**) available at https://digitalcollections.universiteitleiden.nl; (**e**) Photo 13 (ref.^5^) is reproduced with the permission of Balai Pelestarian Cagar Budaya Sulawesi Selatan; (**f**) C. F. O. Ramadhani.

Figure 5 shows new photographic evidence that reiterates the antiquity of the limestone exfoliation. At Leang Sampeang, an anthropomorphic motif in the Austronesian-style clearly overlaps exfoliated areas. The motif is painted directly on a case-hardened limestone surface that has been damaged by at least two phases of exfoliation. The artwork avoids what appears to be an older (weathered and etched) spall-scar and overlaps a younger (fresher) phase of exfoliation. The superposition constraint confirms that at least some of the younger exfoliation phase is older than the artwork, and that the weathered phase is likely to be even older. Therefore, the bulk of the exfoliation within this rock art panel at Leang Sampeang could be hundreds (if not thousands) of years old.

Here, we explore the possibility that the early onset of gypsum efflorescence in the Maros-Pangkep rock art shelters is due to human agency. An important clue to the underlying cause was provided by Suhartono^10^ and Thosibo et al.^12^, and more recently Huntley et al.^15^, who showed that elevated sulphur abundances associated with gypsum are common throughout the rock art panels. Sulphur is the limiting element for gypsum formation. In urban settings, for example, the link between the rise in sulphur air pollution (SO_2_) during the industrial revolution and gypsum formation on carbonate building materials (e.g. limestone) is well documented^16–20^. About 5-10% of the SO_2_ produced by the combustion of fossil fuels (coal, oil) is converted to SO_3_ in the atmosphere and on carbonate surfaces^16^. When dissolved in water, SO_3_ produces sulphuric acid (H_2_SO_4_) that reacts with carbonate materials to produce gypsum. Before the industrial revolution, urban air pollution originated primarily from the combustion of wood for cooking and heating. The sulphur content of wood (0.05-0.2%) is less than coal (2-3%)^36^, but it has been shown that pre-industrial concentrations of wood-fired air-borne SO_2_ were sufficient to trigger the formation of gypsum on ancient carbonate building facades in Europe^20,37^.

Along these lines, biomass burning associated with the rise of Austronesian rice agriculture ~3,500 years ago^35,38^ would have introduced an influential new source of air-borne sulphur to the Maros-Pangkep rock art. Once established, open-field burning of rice straw would have been inextricably linked to the maintenance of rice agriculture^39,40^. Rice straw residues generally contain 0.1-0.2% sulphur and about 40-60% of it is lost during burning^41,42^. Low temperature rice straw burning (<500°C) can lead to the direct formation of SO_3_ (ref.^43^) and trace elements, such as iron in soot and particulate matter, catalyse the oxidation of SO_2_ to SO_3_ (ref.^16^). Soot is particularly harmful because it serves as a medium for SO_2_ absorption, which reinforces gypsum formation^19,20^. Recent work at Alero Cachaco rock shelter (Chile) identified a close link between layers of soot and gypsum deposited on the walls and ceiling from repeated domestic fire-use over the last ~3,000 years^44^.

Under this scenario, artwork in rock shelters that are well-suited for domestic fire-use would tend be the most vulnerable. The earliest evidence for domestic fire-use in the Maros-Pangkep karst comes from Leang Bulu Bettue, where excavations revealed a close association of human skeletal remains (*Homo sapiens*) and fragments of burnt animal bones dating to 25-16 ka (ref.^45^). Also, the negative hand stencils in the Maros-Pangkep rock shelters are thought to indicate collective ‘ownership’ of an ancient dwelling place by an extended family group^46^. Thus the more than 1,000 hand stencils identified so far^7^ probably mark recurrent occupations and fire-use by many generations of family groups. A related point is that, in addition to releasing sulphur, repeated heating of cave walls and ceilings by fire (followed by cooling) can create micro-fractures in limestone^47,48^. This pre-conditioning would facilitate gypsum growth beneath case-hardened surfaces that have been breached. If this is the case, the link between human fire-use, air-borne sulphur and gypsum efflorescence could extend back to ~40 ka, the age of the earliest dated human hand stencil in the Maros karst^1^.

It is important to note that most of the Maros-Pangkep rock shelters are adjacent to open-field burning around the periphery of the karst, and are thus prime locations for gypsum formation (Supplementary Fig. 2). Gypsum crystals are soluble in water and can only take hold on protected surfaces that are not directly exposed to rain, like caves and shelters^49^. Dry deposition of sulphur bearing acidic gases and soot react with damp limestone surfaces to activate gypsum formation^50^. The efficacy of anthropogenic SO_2_ pollution can be enhanced in cave settings where biofilms aid moisture retention and facilitate salt penetration beneath limestone surfaces^51^. Once established, the large diurnal range in temperature (~10 °C) and relative humidity at Maros-Pangkep is ideal for gypsum crystals to shrink and swell and damage rock art.

Taken together, the long and varied histories for the exposure of Maros-Pangkep rock art to agricultural and domestic fire-use may explain the differences in surface exfoliation among shelters. The cumulative degradation of artwork should reflect the concentration of anthropogenic sulphur, exposure history, and extent to which the limestone has been pre-conditioned for gypsum growth by domestic fire-use. The legacy of this millennial-scale history of human fire-use was obvious in the mid-1800s. During his journeys from Makassar to the Maros karst in the dry-seasons of 1856 and 1857, Alfred Russel Wallace^52^ noted “*The country was at first a uniform plain of burnt-up rice-grounds, but at a few miles’ distance precipitous hills appeared …… but owing to the perpetual haze over the land at this time of year, I could nowhere discern the high central range of the peninsula* …..” (p. 219, 237).

### Has the loss of rock art accelerated in recent decades?

There are no systematic observations to show that rock art panel loss in the Maros-Pangkep occurred primarily in recent decades, or that the rate of loss is accelerating. However, historical photographs by the early archaeologist H. R. van Heekeren provide definitive evidence for advanced exfoliation of the rock art by 1950 (refs.^4,53^). Damaged artwork was documented in three limestone shelters in February-April 1950 during the course of shallow excavations in Leang Pettae that encountered evidence for domestic fire-use (ash layers, charcoal and partially calcined bones). The condition of negative hand stencils on red background pigment discovered in Leang Pettae on 26 February 1950 was as follows: “*The red mineral has peeled off in several places and has caused the loss of many details*” (ref.^53^, p. 95). On 5 March 1950, poorly preserved hand stencils were noted at Leang Burung about 6 km southwest of Leang Pettae: “*On the ceiling near the entrance we discovered traces of several hand-stencils, but the red paint appeared in such a state of weathering and was so blistered, that the stencils were hardly recognizable* …..” (p. 96). “Blistering” of hand stencils also was evident in 1950 in the Leang Jarie rock shelter, ~12 km south of Leang Pettae.

**Figure 6:**
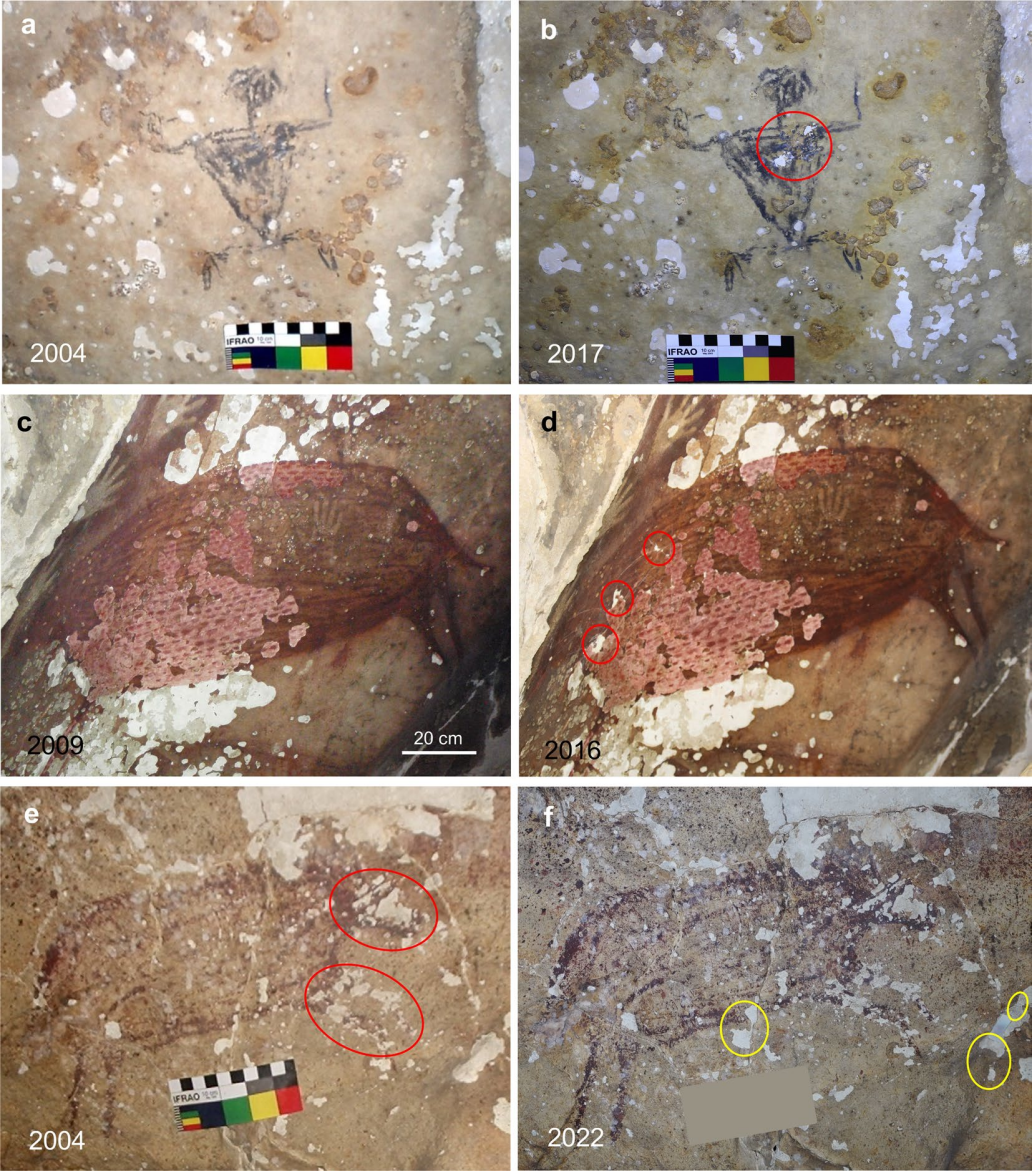
Time-lapse comparisons of rock art exfoliation and vandalism in the Maros karst. (**a**,**b**) Identical exfoliation patterns at Leang Sampeang on 28 September 2004 and 16 July 2017. The restriction of two recent spots of panel loss to the anthropomorph motif (red oval), and their ragged edges, indicates they are due to vandalism (see Supplementary Fig. 7). (**c**,**d**) Evidence for vandalism in Leang Petta Kere. Three patches of panel loss (red ovals) occurred on a pig motif between 15 July 2009 and 3 February 2016 in the absence of change elsewhere in the panel (see Supplementary Fig. 8 for scratch marks confirming vandalism). (**e**,**f**) Leang Pettae on 27 September 2004 and 31 March 2022. Only fine-scale changes in exfoliation have occurred near fracture lines (yellow ovals). The concentration of inactive damage on the head and curved fore-legs of the pig motif (red ovals) indicates it could be due to vandalism before 2004 (see Supplementary Fig. 9). Photo credits: (**a**,**e**) R. C. E. Permana; (**b**,**f**) R. Lebe; (**c**) D. Zwartz; (**d**) H. Scott-Gagan.

The historical baseline provided by van Heekeren’s photographs is valuable for establishing how much panel loss was present before 1950. One of the most frequently visited artwork images in Maros-Pangkep is the “leaping boar” in Leang Pettae^4^. Exfoliation of the limestone walls and ceiling around the painting is extensive. Van Heekeren’s photograph of the boar reproduced in Fig. 5 captures a small part of the pre-1950 exfoliation (ref.^53^, Plate 31). Our photograph in 2022 shows more of the extent of the pre-1950 exfoliation beyond the boar (Fig. 5). By comparison, the minor panel loss over the last 72 years is fine-scale, and occurs primarily along fractures traversing the image. Notably, most of the post-1950 damage is concentrated on the head and curved fore-legs of the boar, and is likely to be due to vandalism (see discussion of Fig. 6 below). Elsewhere in Leang Pettae, some new spall-patches developed between 1950 and 2013 near a popular cluster of hand-stencils (Supplementary Fig. 3). The results show that much of the limestone exfoliation affecting Leang Pettae was present before 1950.

It is critical to determine if the rate of rock art damage has accelerated over the last ~70 years. Fortunately, the state of the limestone exfoliation in some of the rock art sites was documented in 1985-86 (refs.^5,6^), and again in 2003-05 (ref.^7^). These historical reference points allow us to estimate the relative rates of rock art panel loss within two shorter and more recent time-intervals; 1985 to 2022 (~37 years) and 2004 to 2022 (~18 years).

In 1985 and 1986, restorations of damaged artwork were carried out in the Leang Petta Kere (Maros) and Leang Sumpang Bita (Pangkep) shelters^5,6^. Our focus here is the work performed on a heavily exfoliated Pleistocene-style painting of a pig in Leang Petta Kere (Fig. 5, Supplementary Fig. 4). The precise pattern and sizes of the spall-patches that traversed the image in 1985 were photographed prior to its reconstruction. Our photograph in 2021 shows the virtually unaltered state of the exfoliation pattern ~37 years later. A key point is that the boundaries of the spall-patches, both treated and untreated, are still clearly contiguous across the boundary of the artwork. The result shows that, with the exception of one small patch, the exfoliated areas have been inactive since 1985.

A fortuitous photographic record of hand stencil artwork in 36 of the Maros-Pangkep shelters in 2004 offers another valuable reference point^7^. Our up-to-date photographs of artwork panels in nine different shelters, six in Maros and three in Pangkep, show that only minor exfoliation has occurred since 2004 across a total surface area of ~5.8 m^2^ (Fig. 6, Supplementary Figs. 5 and 6). Six of the panels (~4.8 m^2^) show no perceptible exfoliation. However, the occasional appearance of damage specific to the rock art images, in the absence of change elsewhere on the panel, is a prime indicator of vandalism. For example, the time-lapse photography confirms that panel loss around the Austronesian-style anthropomorph image at Leang Sampeang has been inactive, to the finest detail, over the last 18 years. And, remarkably, there has been no discernible loss specific to the ancient motif itself between the time it was painted and 2004. Then, two patches of charcoal pigment were suddenly lost from the chest area of the figure between 2004 and 2017 (Fig. 6). The presence of scratch marks near the patches, and their ragged edges, confirm that the image was targeted by vandals (Supplementary Fig. 7). The presence of scratch marks also confirms that three patches of pigment were directly lost from artwork in Leang Petta Kere due to human agency between 2009 and 2016 (Fig. 6, Supplementary Fig. 8).

In some cases, time-lapse photography also allows the indelible imprint of vandalism to be distinguished within the gradually changing context of chemical exfoliation. For example, the pattern of pigment loss concentrated on the head and curved fore-legs of the “leaping boar” in Leang Pettae barely changed since 2004 (Fig. 6, Supplementary Fig. 9). In contrast, some fine-scale extensions to pre-existing spall-patches occurred between 2004 and 2022 near fractures traversing the panel. The entrance to Leang Pettae was securely gated to prevent unauthorised entry with the establishment of Leang-leang Prehistoric Park in 1999. The added protection might explain the lack of further pigment loss from the head and fore-legs of the boar since 2004.

**Figure 7:**
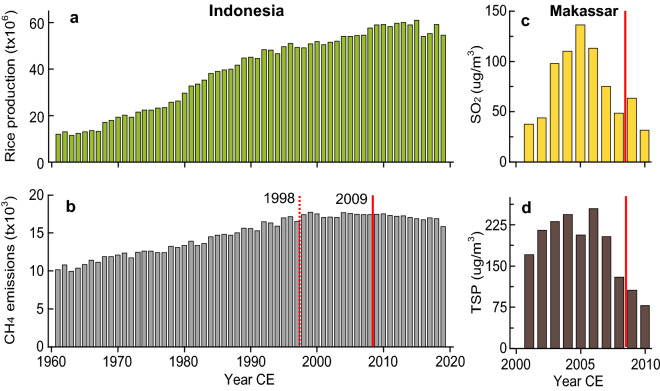
Rice production and rice straw burning in Indonesia and air pollution in the Maros district. (**a**) Rice production in Indonesia. (**b**) Methane (CH_4_) emissions in Indonesia due to rice straw burning (see ref.^66^ for background). Solid red line at 2009 marks the onset of Indonesia Act No 32/2009 to ban agricultural burning; dashed red line marks slowing of rice straw burning after the 1997/98 mega-fires in Indonesia. (**c**,**d**) Sulphur dioxide (SO_2_) concentrations and total suspended particulate (TSP) concentrations in air in Makassar for 2001–2010 (ref.^56^). Rice straw burning and local air pollution were on the decline during the last decade.

To sum-up, the available time-lapse photography indicates that the bulk of the limestone exfoliation in the Maros-Pangkep rock art shelters was present before 1950, and that the rate of change since 1950 could be on the decline. This course of events is generally consistent with the history of in-cave fire-use and rice straw burning around the Maros-Pangkep karst. Domestic in-cave fire-use was undoubtedly on the decline, if not absent, by 1950. On the other hand, rice production in Indonesia (and South Sulawesi) increased substantially after 1950 (Fig. 7). The accompanying increase in rice straw burning led to a significant increase in SO_2_ air pollution and particulate emissions in Indonesia^54^.

It is important to note that the upward trajectory of rice straw burning in Indonesia has plateaued, and even started to reverse, over the last two decades (Fig. 7). The plateauing after ~1998 may have been due, in part, to efforts to curtail biomass burning in the aftermath of the 1997/98 mega-fires in Indonesia^55^. A notable turning point occurred in 2009 with the issuance of Act No 32/2009 on Environmental Protection and Management, which curtailed agricultural burning. Biomass burning during the dry season (July–September) has been a major factor contributing to high SO_2_ and particulate concentrations in air in the city of Makassar on the Maros coastal plain^56,57^. However, SO_2_ and particulate air pollution in Makassar both show distinct downward trends starting around 2005 suggesting that the Maros-Pangkep rock art may already be benefitting from waning fire-use.

### Identification of rock art degradation ‘hot-spots’

The results at hand highlight a genuine need to determine which of the Maros-Pangkep art galleries are actively under threat. A reasonably attractive scenario for conservation management would be one where only relatively minor ‘hot-spots’ of panel loss within particular shelters are currently active. In that case, targeted conservation measures could be deployed precisely where required. In this regard, the potential for localised exposure of artwork to vandalism, sulphur emissions from diesel-powered traffic and reactive sulphur-rich cement dust needs to be considered.

#### Vandalism

The extent to which direct human interference has disfigured artwork in the Maros-Pangkep karst may have been under-estimated. Population growth, commercial development and burgeoning tourism in the Maros-Pangkep districts all raise the risk of vandalism to readily accessible rock art. Figure 6 showed that well-known artworks in Leang Petta Kere and Leang Pettae have been degraded by vandalism. And at least one motif in Leang Sampeang, which is accessible from a popular road, also has been damaged.

Time-lapse photography of rock art images set within the broader context of panel loss will be important to distinguish vandalism from chemical exfoliation. A key indicator of human interference is the sudden appearance of pigment loss specific to the artwork in the absence of change elsewhere on the panel. Ragged edges on the damaged area and associated scratch-marks may provide confirmation of human agency. Rock art galleries with vandalism hot-spots that emerge in time-lapse photography could be prioritised for protection by targeted deterrents.

#### Diesel-powered traffic

**Figure 8:**
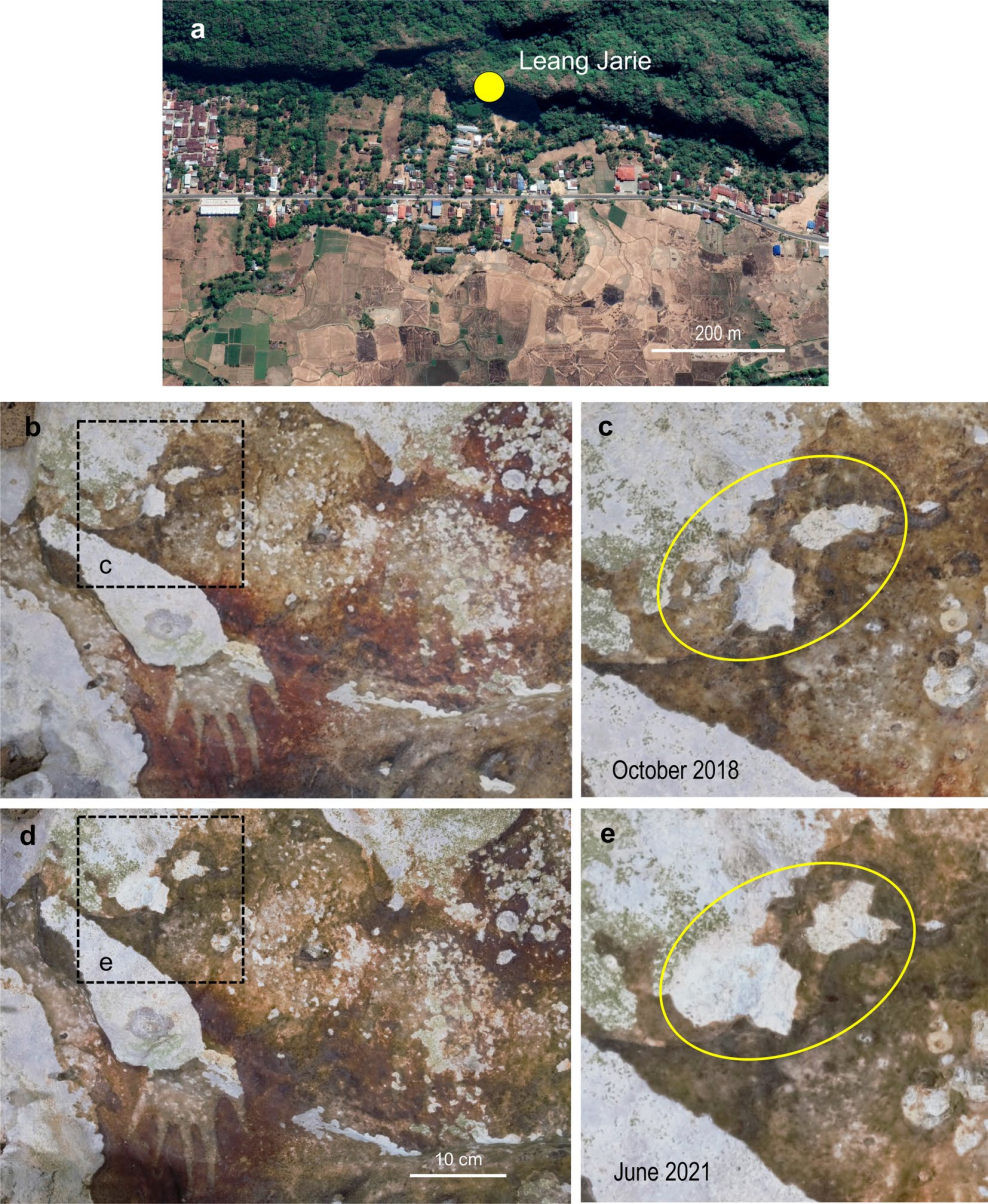
Time-lapse photography of active exfoliation ‘hot-spots’ at Leang Jarie. (**a**) Map showing proximity of the Leang Jarie cave site to rice cultivation, main road traffic and cement-based infrastructure. (**b**,**c**) Overview of inactive exfoliation and close-up of two active spall-scar patches on 8 October 2018. (**d**,**e**) As for (**b**,**c**) but on 30 June 2021. The area covered by the two patches within the yellow oval has almost doubled in ~ 2.5 years. Map image is for 16 August 2019 on Google Earth Pro 7.3.4.8573 (24 March 2022) at http://www.google.com/earth/index.html (accessed 1 May 2022). Photo credits: R. Lebe.

Gypsum efflorescence caused by sulphur emissions from diesel-powered traffic should be considered where hot-spot panel loss, either within or outside of artworks, is detected in proximity to roads (Fig. 8). Development on the coastal plain adjacent to the Maros-Pangkep karst has resulted in an increase in road transport of goods, trans-Sulawesian travellers and tourists. As of 2008, more than 600,000 tourists per year are transported along secondary roads in proximity to rock art sites within Bantimurung-Bulusaraung National Park. The sulphur content of diesel fuel in Indonesia is decreasing, but mostly remains relatively high (up to 0.25%), leaving the artwork at risk of exposure to SO_2_ and acidic soot.

A case in point is the Leang Jarie shelter, which is sign-posted on a main road between Makassar and central Sulawesi that passes within ~200 m of the site (Fig. 8). Here, time-lapse photography conducted since October 2018 has identified an active exfoliation hot-spot within a rock art panel dominated by inactive spall-patches^3^. The width of the most active spall-patch under investigation has doubled from ~2.5 cm to ~5 cm between October 2018 and June 2021.

#### Cement dust

The expansion of limestone mining for cement production facilities along the periphery of the Maros-Pangkep karst poses additional threats to rock art^14,15,58^. Aside from the potential for physical damage, cement manufacturing facilities release SO_2_ and cement dust to the surrounding atmosphere. Sulphur dioxide emissions from cement factories are primarily related to the coal-fired kiln system that heats raw materials (limestone and clay) to form clinker. Perhaps of more concern, however, is that the production of Portland cement, the basic ingredient of concrete, requires the addition of 3-5% gypsum to regulate its setting time^59^. The main source of sulphur-rich dust is the stacks of the kiln system, but emissions can occur during grinding processes and may arise from storage and handling of finely ground materials^60^. Therefore, it is possible that powdery cement dust, with high SO_3_ content, could be adsorbed onto damp limestone surfaces in nearby rock shelters and react to form gypsum^61^.

A key point here, however, is that cement dust released during the construction of domestic and commercial infrastructure in recent decades may have posed a more insidious risk to the Maros-Pangkep artwork. Portland cement is used in virtually all concrete structures and, in the Maros-Pangkep districts, is generally mixed on-site for the construction of buildings, walls, culverts and, in some cases, secondary roads. Chemically reactive sulphur-rich dust released during the concreting process could act as a catalyst for gypsum formation. Again, a potential target is the Leang Jarie shelter, which is adjacent to a ~200 m wide strip of land with cement-based infrastructure between a main road and the edge of the karst (Fig. 8). In this case, the active hot-spot panel loss observed today could reflect, in part, the chemical mobilisation of cement-dust sulphur that has accumulated in the interstitial porosity of the limestone bedrock.

## Conclusions

Our findings show that there are grounds for being cautiously optimistic that most of the limestone exfoliation in the Maros-Pangkep rock art shelters is currently inactive. The available time-lapse photography of rock art panel loss from 1950 to 2022 raises the possibility that the rate of rock art exfoliation might even be on the decline. The pattern is consistent with the history of fire-use in southwest Sulawesi and air-borne sulphur as the primary agent driving gypsum efflorescence and rock art degradation over hundreds and perhaps thousands of years.

There is an urgent need to determine which artworks are actively under threat. Time-lapse photography will be pivotal for assessing the status of individual rock shelters. The results presented here show that evidence for limestone exfoliation hot-spots, where present, should emerge within the broader context of pre-existing rock art panel loss. Locating historical photographs of the Maros-Pangkep artwork will be essential to evaluate the risk posed by hot-spot activity revealed through systematic observations. Active areas that do arise could be prioritised for targeted monitoring, mitigation and conservation measures to ensure the longevity of these artistic treasures for future generations.

## Methods

### SST reconstruction

The glacial-to-Holocene SST reconstructions for Makassar Strait in Fig. 2 are based on Mg/Ca-SST data for core MD9821-62 (4° 41’ S, 117° 54’ E, 1855 m depth; (ref.^21^) and core 70GGC (3° 34’ S, 119° 23’ E, 482 m depth; (ref.^22^) at 200-400 year resolution. Briefly, Mg/Ca was measured on tests of the planktonic foraminifer *Globigerinoides ruber*, which lives in the surface mixed layer, and converted to SST using calibration relationships appropriate for Makassar Strait. Age models for the records are based on AMS ^14^C dates on planktonic foraminifera with ^14^C reservoir corrections of 400-500 years subtracted from the dates before they were converted to kyrBP (thousand years before present, where present is 1950 CE). A broad network of *G. ruber* Mg/Ca-based SST reconstructions defines a consistent ~3-4 °C increase in glacial-to-Holocene SST across the western sector of the Indo-Pacific Warm Pool^22,23^.

### Summer monsoon rainfall reconstruction

The ~40-kyr record of austral summer monsoon rainfall in Fig. 2 is based on δ^18^O data at ~50-year resolution for stalagmites GB09-3 and GB11-9 from Gempa Bumi Cave (5° 01’ S, 119° 40’ E; ~140 m above sea level) in the Maros karst^24^. The age models for the records are based on 44 U-Th dates (converted to kyrBP). An ice volume correction has been applied to the stalagmite δ^18^O values to account for the effect of the 1‰ decrease in seawater δ^18^O across the glacial-Holocene transition^24^. The adjusted δ^18^O record is interpreted in terms of relative changes in monsoon rainfall on the premise that the δ^18^O of tropical rainfall is inversely proportional to rainfall amount. Measurements of Mg/Ca in stalagmites from the Maros karst have validated the large change in δ^18^O between drier glacials and wetter interglacials in southwest Sulawesi^62^.

### Instrumental records of rainfall

Figure 3 shows monthly rainfall totals for the study area from the Global Precipitation Climatology Center (GPCC). The GPCC rainfall data are derived from quality controlled rain-gauge stations with historical and Global Telecommunication System data, and interpolated onto a latitudinal-longitudinal grid^63^. The record in Fig. 3 is based on rainfall data available for January 1891 to December 2019 for the 0.25° x 0.25° grid-square centred on 5.125° S, 119.625° E (covering the coastal plain between Makassar and Maros).

The GPCC rainfall time-series are not bias corrected for systematic gauge measuring errors^63^. Therefore, our presentation of the GPCC record in Fig. 3 is restricted to 1950 to 2019 due to an abrupt offset in the mean (+42 mm) and standard deviation (+33 mm) of the record after 1950 (Supplementary Fig. 1). The accuracy of ENSO-related rainfall variability in the GPCC record after 1950 (our primary interest) was validated using semi-continuous local rainfall data for World Meteorological Organization station 97180 (5.1° S, 119.6° E) available from the Global Historical Climatology Network (GHCNv4)^64^. Supplementary Figure 1 shows good agreement in the interannual variability of rainfall over the 69-year interval of overlap (1950-2018). We note that the monthly totals in the GPCC record are 23 mm higher, on average, than those at WMO station 97180. The difference accounts for 55% of the +42 mm offset in average rainfall in the GPCC record after 1950.

### Assessment of rock art degradation

Figure 4 summarises two assessments of rock art degradation in Maros-Pangkep^7,11^. Permana^7^ assessed 727 hand stencil artworks in 36 cave sites in the Maros-Pangkep karst in July 2003, September 2004 and February 2005. The condition of each hand stencil was assessed as “clear” or “not clear”, based primarily on whether the outline of the stencil was easy to discern. Therefore, the assessments account for any type of damage to the hand stencils, not just exfoliation, and do not extend to the cave site as a whole. We calculated the percentage of hand stencils damaged at each site, with outcomes ranging from 0% (none damaged) to 100% (all damaged) (see Supplementary Table 1).

Mulyadi^11^ assessed the condition of artwork in 44 cave sites in November-December 2013, including 21 sites previously examined by Permana^7^. The general status of each site was assigned one of three rankings; “good”, “moderate” or “poor” based on the level of damage due to physical weathering (cracked, broken, worn out), biological weathering (growth of algae, moss, lichen) and chemical weathering (salt damage, cementation)^11^. There is a notable difference in the frequency of severe damage based on the two classification systems: Permana^7^ noted only one site in 2003-2005 where all hand stencils were damaged whereas in 2013 Mulyadi^11^ noted 12 severely damaged sites.

## Supplementary Information

Supplementary Information.

## Data Availability

All data generated or analysed for the current study are included in the article (and its Supplementary Information file), or are available from refs.^7,11^ (rock art assessments), ref.^24^ (δ^18^O), ref.^56^ (SO_2_, TSP) and the following websites: Mg/Ca-SST for ref.^21^ (https://pangaea.de) and ref.^22^ (https://www.ncei.noaa.gov/products/paleoclimatology); rainfall for ref.^63^ (https://opendata.dwd.de/climate_environment/GPCC/html/fulldata-monthly_v2020_doi_download.html) and ref.^64^ (https://www.ncei.noaa.gov/data/ghcnm/v4beta/access/); the SOI (http://www.bom.gov.au); rice production (https://www.fao.org/faostat/en/#data/QCL) and rice residue CH_4_ emissions (https://knoema.com/faoemagcr2017/burning-crop-residues-1961-2050).
