# Supplementary material for: The historical impact of anthropogenic air-borne sulphur on the Pleistocene rock art of Sulawesi
Source: Sci Rep. 2022 Dec 13;12:21512. doi: 10.1038/s41598-022-25810-1 (PMC9748042; doi:10.1038/s41598-022-25810-1)
Supplement: Supplementary file 1 — Supplementary Information. [file 41598_2022_25810_MOESM1_ESM.pdf]

Supplementary Information

The historical impact of anthropogenic air-borne sulphur on the Pleistocene rock art of Sulawesi

Michael K. Gagan[1,2,3], Halmar Halide[4], Raden Cecep Eka Permana[5], Rustan Lebe[6], Gavin B. Dunbar[7], Alena K. Kimbrough[1], Heather Scott-Gagan[3], Dan Zwartz[8], Wahyoe S. Hantoro[9,1]

[1]School of Earth, Atmospheric and Life Sciences, University of Wollongong, Wollongong, NSW 2522, Australia
[2]School of Earth and Environmental Sciences, The University of Queensland, St Lucia, QLD 4072, Australia
[3]Australasian Earth Systems, P.O. Box 1290, Canberra, ACT 2601, Australia
[4]Departemen Geofisika, Fakultas Matematika dan Ilmu Pengetahuan Alam, Universitas Hasanuddin, Makassar 90245, Indonesia
[5]Departemen Arkeologi, Fakultas Ilmu Pengetahuan Budaya, Universitas Indonesia, Depok 16424, Indonesia
[6]Balai Pelestarian Cagar Budaya Sulawesi Selatan, Makassar 90111, Indonesia
[7]Antarctic Research Centre, Victoria University of Wellington, Wellington 6140, New Zealand
[8]Centre for Science and Society, Victoria University of Wellington, Wellington 6140, New Zealand
[9]Research Center for Geotechnology, Indonesian Institute of Sciences, Bandung 40135, Indonesia

Supplementary Figures

Figure 1: Comparison of rainfall datasets for the study area.

Figure 2: Location and status of rock art sites shown in the main figures and Supplementary figures.

Figure 3: Historical photographs of rock art exfoliation at Leang Pettae.

Figure 4: Restoration of exfoliated rock art in Leang Petta Kere.

Figure 5: Time-lapse comparisons of exfoliation in three Pangkep rock art shelters.

Figure 6: Time-lapse comparisons of exfoliation in three Maros rock art shelters.

Figure 7: Example of rock art vandalism at Leang Sampeang.

Figure 8: Example of rock art vandalism at Leang Petta Kere.

Figure 9: Example of possible rock art vandalism at Leang Pettae.

Supplementary Data

Table 1: Results of rock art damage assessments in the Maros-Pangkep karst.

Supplementary References

Supplementary Figures

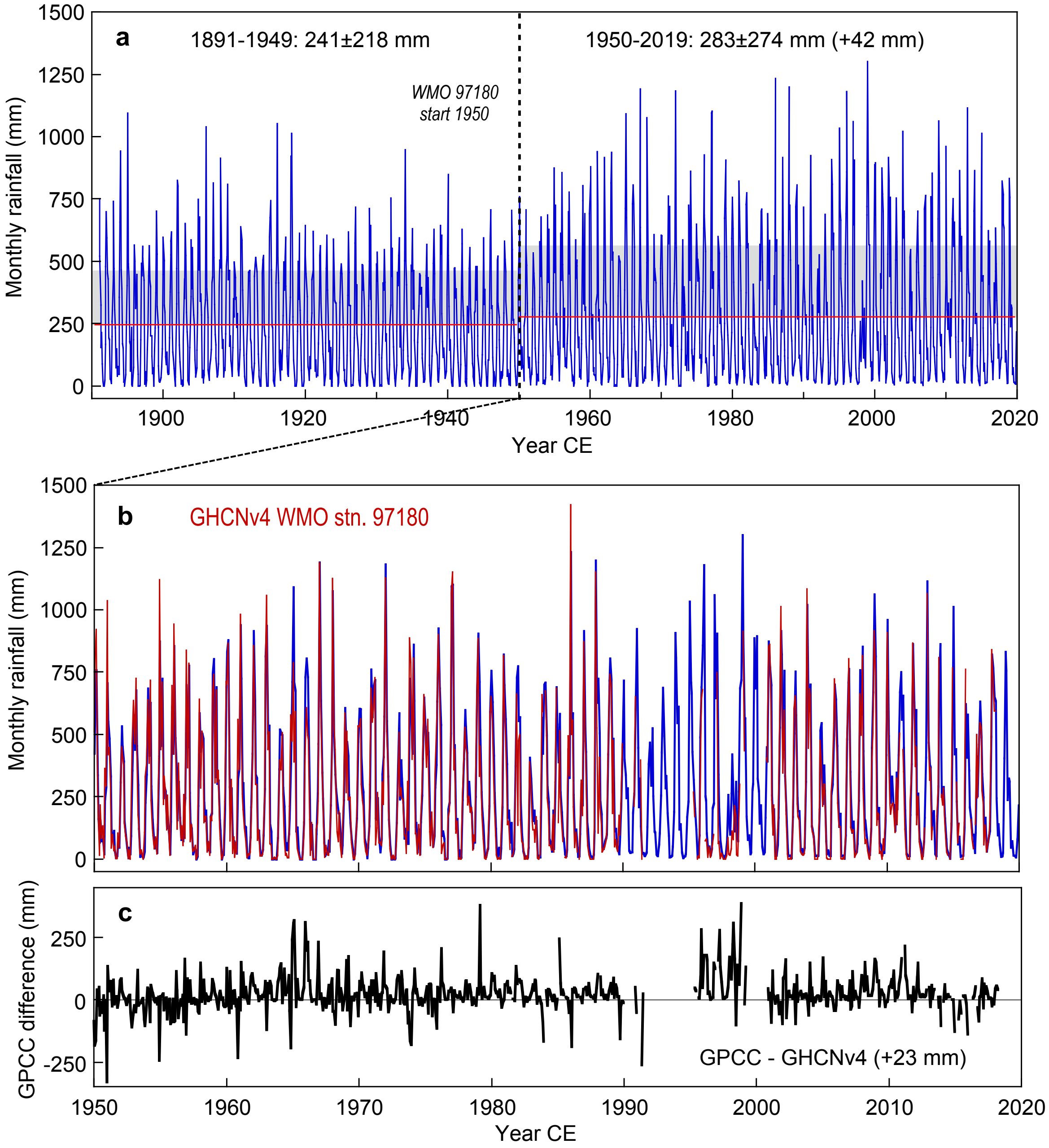

Supplementary Figure 1 | Comparison of rainfall datasets for the study area. (**a**) Monthly rainfall totals for 1891-2019 for the Global Precipitation Climatology Center (GPCC) 0.25° x 0.25° grid-square centred on 5.125° S, 119.625° E (Schneider et al., 2020). There is an offset in the mean (+42 mm) and standard deviation (+33 mm) of the record after 1950. (**b**) Comparison of the GPCC rainfall record with the discontinuous record for World Meteorological Station 97180 (5.1° S, 119.6° E) available at the Global Historical Climatology Network (GHCNv4, Lawrimore et al., 2011). The good agreement of the GPCC and GHCNv4 records after 1950 validates the use of the continuous GPCC record for the purposes of examining ENSO variability in Figure 3. (**c**) Differences between the GPCC and GHCNv4 rainfall records. The GPCC record is 23 mm higher than the GHCNv4 record, on average, which accounts for 55% of the +42 mm offset in GPCC rainfall after 1950. Rainfall data are from: https://opendata.dwd.de/climate_environment/GPCC/html/fulldata-monthly_v2020_doi_download.html and https://www.ncei.noaa.gov/data/ghcnm/v4beta/access/.

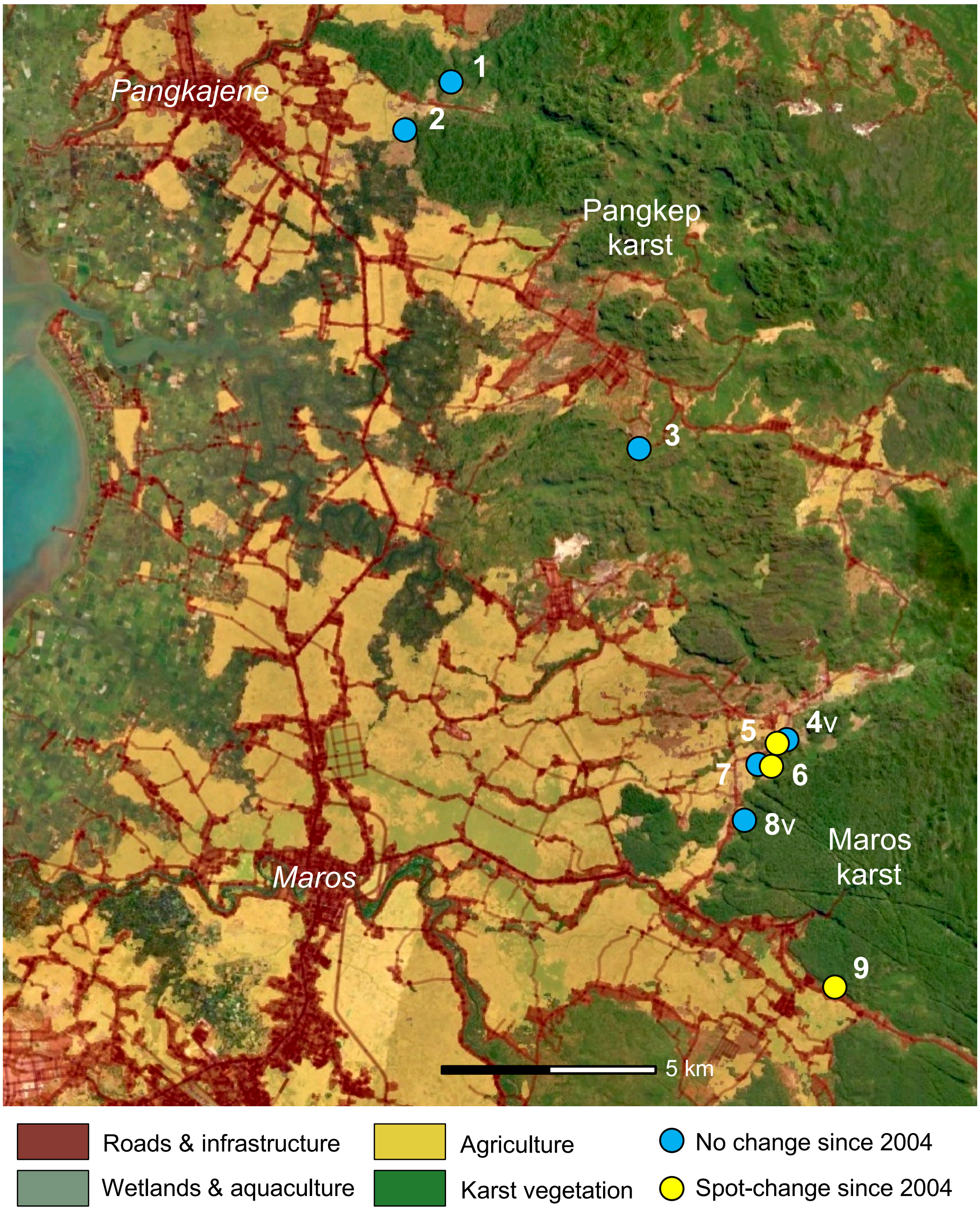

Supplementary Figure 2 | Location and status of rock art sites shown in the main figures and Supplementary figures. Leang Sakapao (1, Fig. S5), Leang Sassang (2, Fig. S5), Leang Sumpang Bita (3, Fig. S5), Leang Petta Kere (4, Fig. 6; Fig. S8), Leang Pettae (5, Fig. 6; Fig. S3), Leang Uluwae (6, Fig. S6), Leang Pajae (7, Fig. S6), Leang Sampeang (8, Fig. 6, Fig. S6, S7), Leang Jarie (9, Fig. 8). The status of rock art exfoliation since 2004 is based on time-lapse photography (see main text figures and Supplementary figures indicated for each site). Site numbers with "v" have rock art panel loss due to vandalism (only) since 2004. The base map was created by A. K. Kimbrough in QGIS 3.20 (https://qgis.org/en/site/) using the Bing Virtual Earth base map (2010 Microsoft Corporation and its data suppliers) overlayed by the Sulawesi roads dataset from Humanitarian Open Street Maps (https://data.humdata.org/dataset/hotosm_idn_sulawesi_roads) and the 2021 ESRI Sentinel-2 10m Land Use/Land Cover data from Impact Observatory, Microsoft and Esri (https://env1.arcgis.com/arcgis/rest/services/Sentinel2_10m_LandCover/ImageServer). Site location co-ordinates are from Balai Pelestarian Cagar Budaya Sulawesi Selatan.

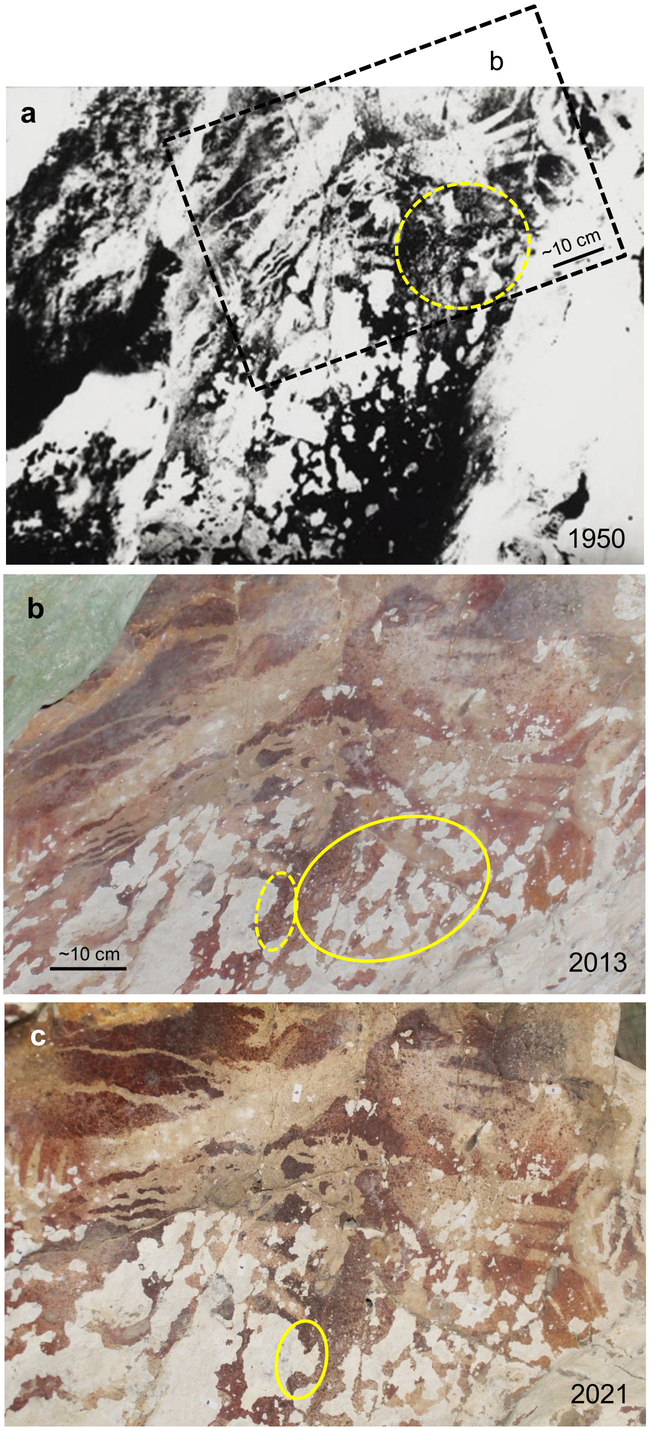

Supplementary Figure 3 | Historical photographs of rock art exfoliation at Leang Pettae. (**a**) Extent of exfoliation in hand stencil panel in 1950 (van Heekeren, 1952). (**b**) Minor additions to the exfoliation occurred between 1950 and 18 June 2013 (exfoliation within solid yellow oval has increased relative to exfoliation within dashed yellow oval in panel a). (**c**) There was little change in the area of exfoliation during the ~8.5 years between 18 June 2013 and 26 December 2021 (exfoliation within yellow oval has increased slightly). Photo credits: (a) Photo for 1950 is available at https://digitalcollections.universiteitleiden.nl. Recent photographs by G. B. Dunbar (b) and M. Syafrizal (c).

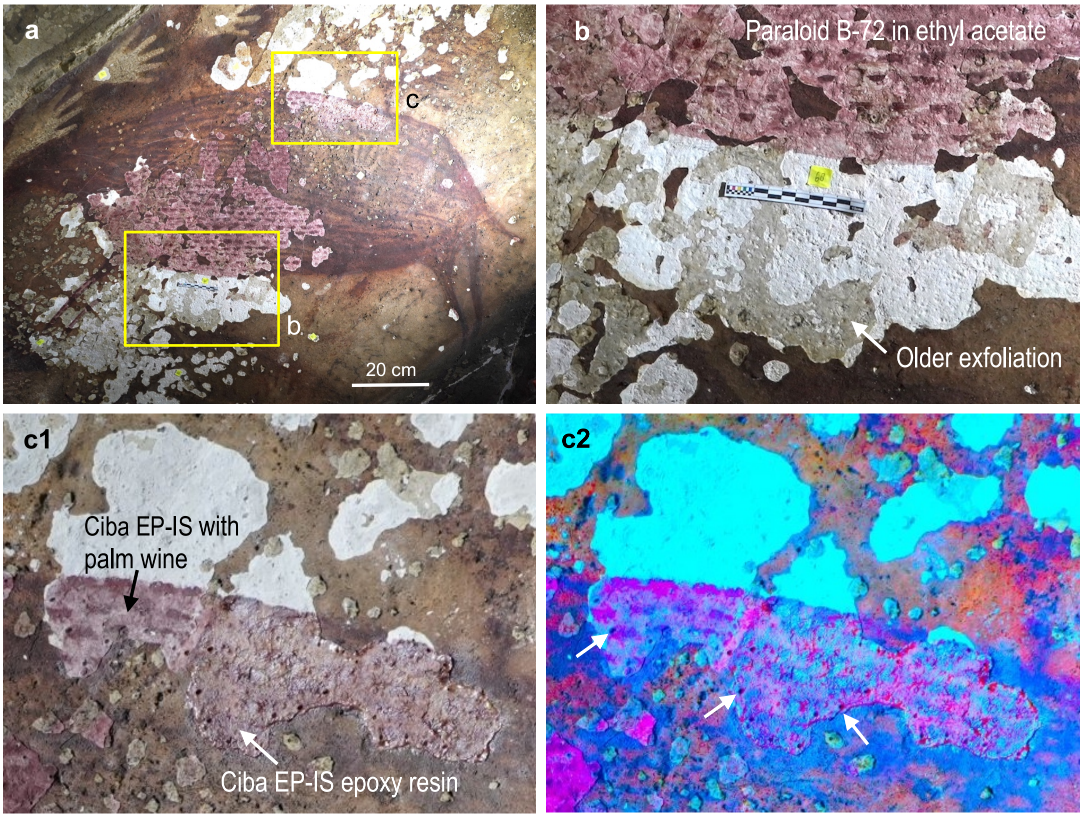

Supplementary Figure 4 | Restoration of exfoliated rock art in Leang Petta Kere (Samidi, 1985; Samidi, 1986). (**a**) Condition of painting of a pig on 9 September 2021 with key restoration areas shown in insets. (**b**) Close-up of two phases of exfoliation treated in 1985-86 with Paraloid B-72 in ethyl acetate solution. (**c1, c2**) Close-ups of exfoliated area treated in 1985-86 with (1) Ciba EP-IS epoxy resin (shiny surface) and (2) Ciba EP-IS with palm wine. White arrows in panel c2 mark examples of 1-1.5 mm diameter respiration holes drilled into exfoliated surfaces to release water trapped within pore spaces. The continuity of the exfoliation across the treated and untreated areas shows that the size of the exfoliation (both treated and untreated) has not changed since 1985-86. Photo credit: C. F. O. Ramadhani. Panel c2 has been enhanced using Lab Color in Adobe Photoshop 22.4.3.

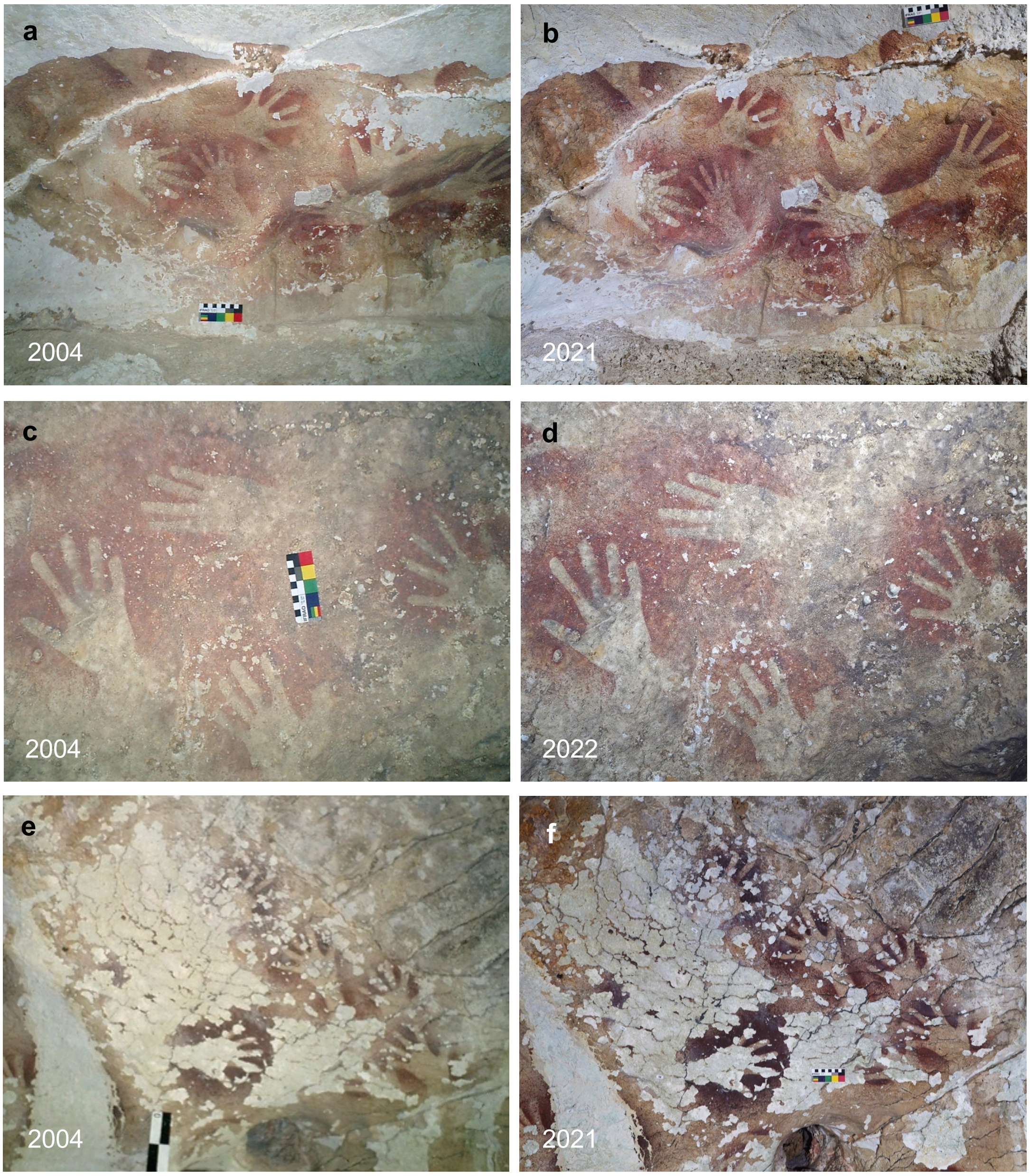

Supplementary Figure 5 | Time-lapse comparisons of exfoliation in three Pangkep rock art shelters. (**a, b**) Identical exfoliation patterns at Leang Sakapao on 25 September 2004 and 27 June 2021. (**c, d**) As for (a, b) but for Leang Sassang on 25 September 2004 and 10 May 2022. (**e, f**) As for (a, b) but for Leang Sumpang Bita on 27 September 2004 and 28 June 2021. The results show that essentially all of the exfoliation in these three Pangkep rock art panels occurred before 2004. Photo credits: (a, c, e) R. C. E. Permana; (b, d, f) R. Lebe.

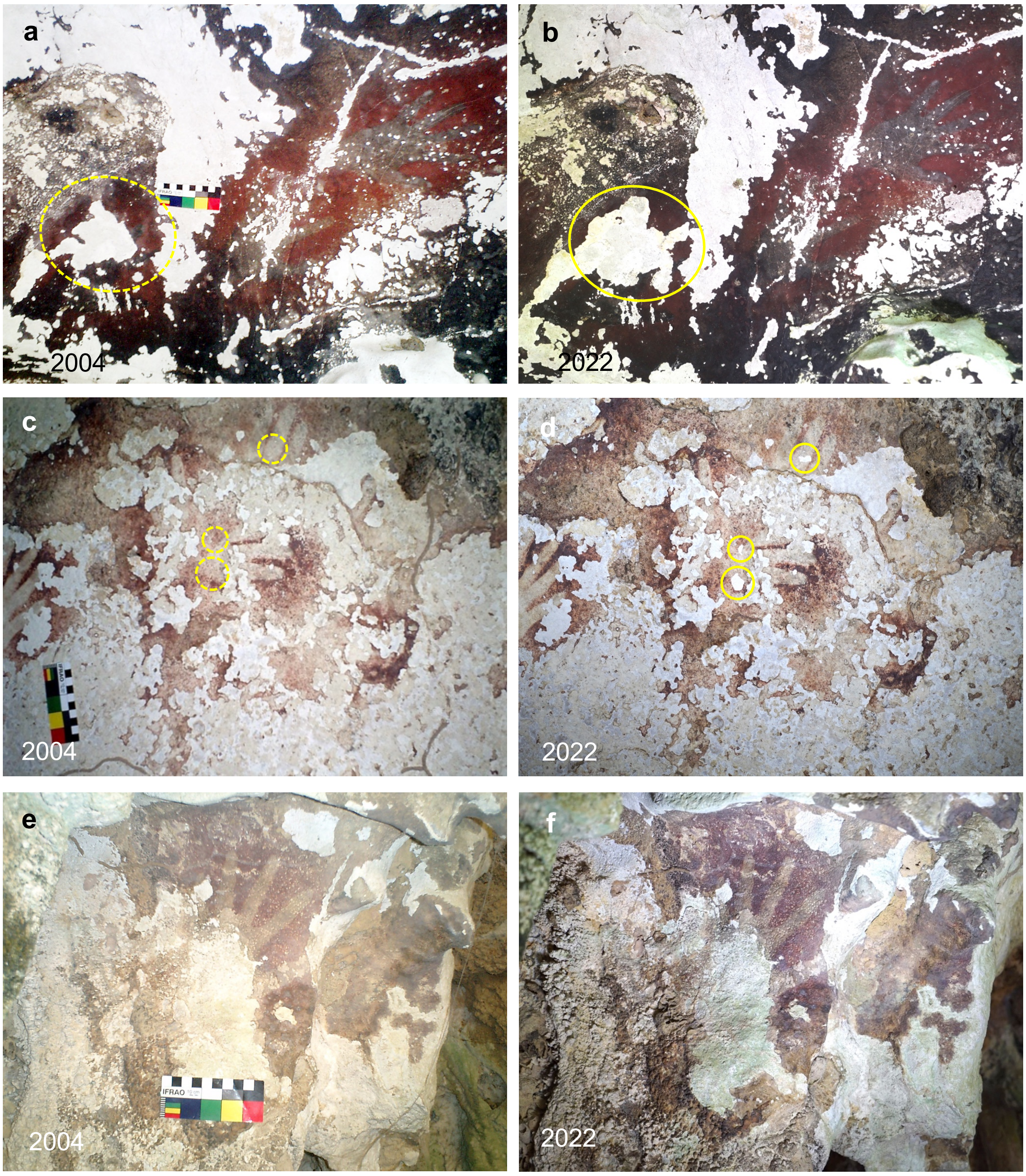

Supplementary Figure 6 | Time-lapse comparisons of exfoliation in three Maros rock art shelters. (**a, b**) Exfoliation patterns at Leang Uluwae on 28 September 2004 and 12 April 2022. Yellow ovals mark area with 'hot-spot' infilling between 2004 and 2022. (**c, d**) As for (a, b) but for Leang Sampeang on 28 September 2004 and 10 May 2022. (**e, f**) As for (a, b) but for Leang Pajae on 28 September 2004 and 12 April 2022. The results show that the bulk of the exfoliation in these three Maros rock art panels occurred before 2004. Photo credits: (a, c, e) R. C. E. Permana; (b, d, f) R. Lebe.

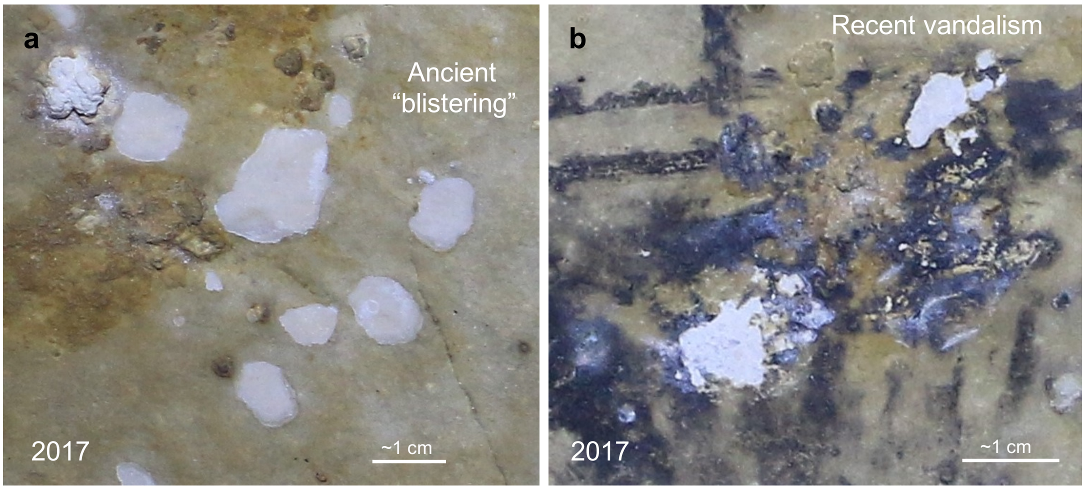

Supplementary Figure 7 | Example of rock art vandalism at Leang Sampeang. (**a**) Close-up of inactive chemically-induced “blistering” on 16 July 2017. (**b**) The same panel showing loss of two patches of charcoal pigment from the Austronesian-style anthropomorph between 28 September 2004 (see main text Fig. 6a, b) and 16 July 2017. The presence of scratch marks near the patches, and their ragged edges, indicate that the image was targeted by vandals between 2004 and 2017. Photo credit: R. Lebe.

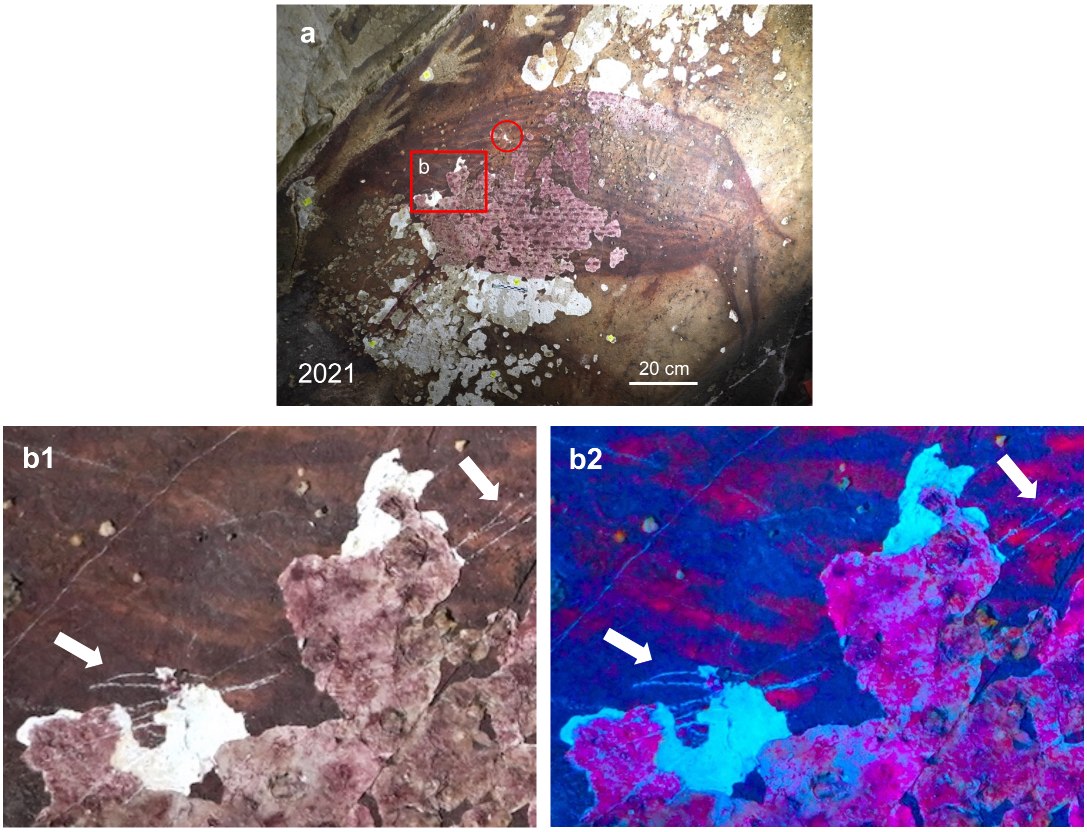

Supplementary Figure 8 | Example of rock art vandalism at Leang Petta Kere. (**a**) Painting of a pig with three patches of panel loss (red highlights) that occurred between 15 July 2009 and 3 February 2016 in the absence of change elsewhere in the panel (see main text Fig. 6c, d). (**b1, b2**) Close-up of the panel loss on 9 September 2021. White arrows indicate associated scratch-marks due to vandalism. Photo credit: C. F. O. Ramadhani. Panel b2 has been enhanced using Lab Color in Adobe Photoshop 22.4.3.

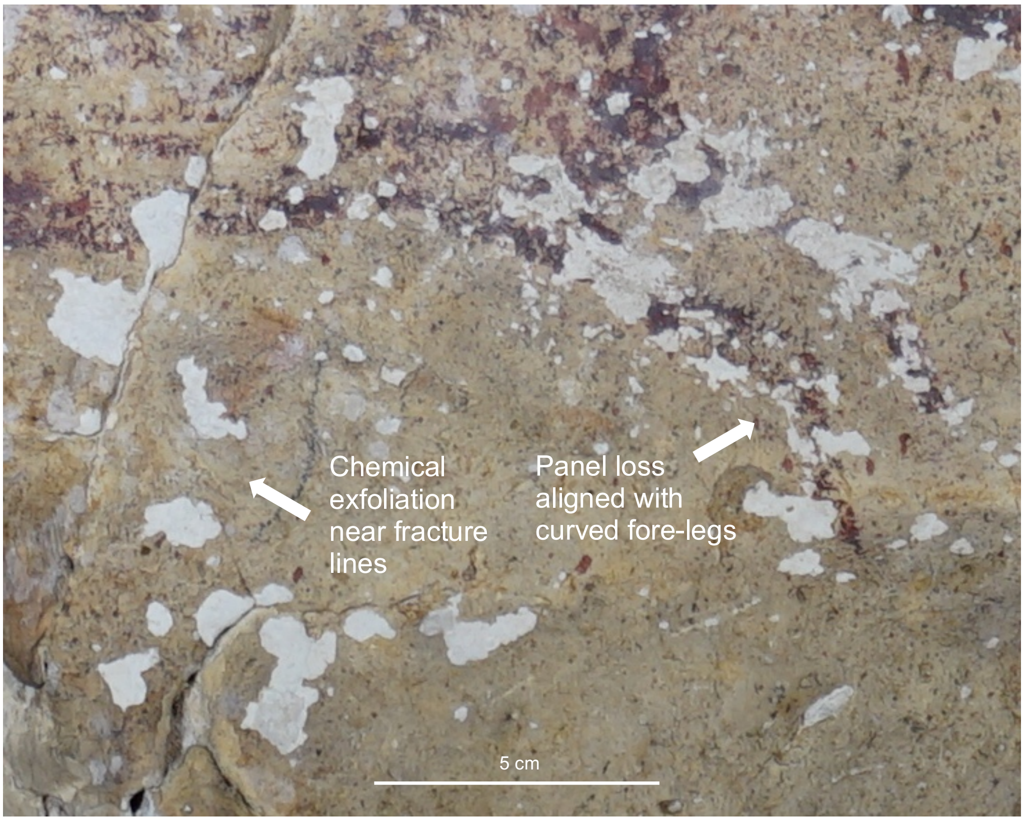

Supplementary Figure 9 | Example of possible rock art vandalism at Leang Pettae. The photograph shows two styles of exfoliation around the painting of the "leaping boar" on 31 March 2022. Chemically-induced patches of exfoliation in proximity to fractures have increased in size since 27 September 2004 (see main text Fig. 6e, f). In contrast, relatively inactive panel loss is concentrated on the curved fore-legs of the boar. The somewhat ragged appearance of the pigment loss, its concentration on the motif itself, and the absence of change since 2004 (see Fig. 6e, f) suggests it is due to targeted vandalism. Photo credit: R. Lebe.

Supplementary Data

Supplementary Table 1. Rock art (hand stencil) damage assessment data from Permana (2008) and Mulyadi (2016). The assessments of Permana (2008) have been converted to the percentage of hand stencils damaged at each site.

Figure 4 ID	Cave site	District	Number assessed	Preserved	Damaged	Damage (%)	Mulyadi (2016)*
1	Leang Pettae Kere (Fig. 5, 6; Fig. S4, S8)	Maros	12	12	0	**0**	*Pettakere*
	Leang Samungkeng II	Maros	3	3	0	**0**	
2	Leang Sakapao (Fig. S5)	Pangkep	24	23	1	**4**	
3	Leang Sumpang Bita (Fig. S5)	Pangkep	81	78	3	**4**	
	Leang Saluka	Pangkep	33	30	3	**9**	
4	Leang Kassi	Pangkep	9	8	1	**11**	
5	Leang Pattenungan	Pangkep	19	17	2	**11**	*Patennung*
	Leang Cumi Lantang	Pangkep	42	37	5	**12**	
6	Leang Lambatorang	Maros	34	30	4	**12**	
	Leang Ellu Loang	Maros	8	7	1	**13**	
7	Leang Tampuang	Maros	71	60	11	**15**	*Tapuang*
8	Leang Cammingkana	Pangkep	36	29	7	**19**	
9	Leang Jing	Maros	30	24	6	**20**	
10	Leang Sassang (Fig. S5)	Pangkep	15	12	3	**20**	
11	Leang Barugayya	Maros	29	23	6	**21**	*Barugae*
	Leang Samungkeng I	Maros	4	3	1	**25**	
12	Leang Ambe Pacco	Maros	10	7	3	**30**	
	Leang Minrallenge	Maros	20	14	6	**30**	
	Leang Garunggung	Pangkep	48	33	15	**31**	
13	Leang Bulu Sumi	Pangkep	8	5	3	**38**	
14	Leang Samungkeng III	Maros	10	6	4	**40**	*Samungkeng*
15	Leang Barattedong	Maros	10	6	4	**40**	*Baratedong*
16	Leang Jarie (Fig. 8)	Maros	17	10	7	**41**	
	Leang Burung VII	Maros	7	4	3	**43**	
17	Leang Lompoa	Pangkep	2	1	1	**50**	
	Leang Burung II	Maros	6	3	3	**50**	
18	Leang Pajae II (Fig. S6)	Maros	6	3	3	**50**	*Pajae*
	Leang Sampeang II	Maros	8	4	4	**50**	
19	Leang Uluwae (Fig. S6)	Maros	8	4	4	**50**	
20	Leang Tengae	Maros	11	5	6	**55**	*Tenggae*
	Leang Kamase	Maros	9	4	5	**56**	
	Leang Timpuseng	Maros	7	3	4	**57**	
21	Leang Batang Lamara	Pangkep	10	4	6	**60**	
	Leang Sampeang I (Fig. 6; S6, S7)	Maros	29	10	19	**66**	
	Leang Lompoa II	Maros	48	16	32	**67**	
	Leang Burung V	Maros	3	0	3	**100**	

8 assessment sites shown in main text figures and supplementary figures (grey)

8 of 10 sites studied by Huntley et al. (2021) (yellow)

* Assessments from Table 2 of Mulyadi (2016). Site names are shown where spelling is slightly different from Permana (2008).

Good preservation (green)

Moderate preservation (orange)

Poor preservation (red)

Supplementary References

Huntley, J. *et al.* The effects of climate change on the Pleistocene rock art of Sulawesi. *Sci. Rep.* **11**, 9883; 10.1038/s41598-021-87923-3 (2021).

Lawrimore, J. H. *et al.* Global Historical Climatology Network - Monthly (GHCN-M) Version 4. NOAA National Centers for Environmental Information. https://doi.org/10.7289/V5X34VDR (2011).

Mulyadi, Y. Kajian keterawatan lukisan gua prasejarah di kawasan karst Maros Pangkep Sulawesi Selatan. *J. Konservasi Cagar Budaya Borobudur* **10**, 15–27 (2016).

Permana, R. C. E. *Pola Gambar Telapak Tangan Pada Gua-gua Prasejarah di Wilayah Pangkep-Maros Sulawesi Selatan*. PhD thesis, Universitas Indonesia (2008).

Samidi. *Laporan hasil survai konservasi lukisan Gua Sumpang Bita dan pelaksanaan konservasi lukisan Gua Pettae Kere.* Proyek pemugaran dan pemeliharaan peninggalan sejarah dan purbakala Sulawesi Selatan (1985).

Samidi. *Laporan konservasi lukisan perahu/sampan di Gua Sumpang Bita (tahap awal) dan konservasi babi rusa di Gua Pettae Kere (penyelesaian).* Proyek pemugaran dan pemeliharaan peninggalan sejarah dan purbakala Sulawesi Selatan (1986).

Schneider, U., Becker, A., Finger, P., Rustemeier, E. & Ziese, M. GPCC full data monthly product version 2020 at 0.25°: monthly land-surface precipitation from rain-gauges built on GTS-based and historical data. https://doi.org/10.5676/DWD_GPCC/FD_M_V2020_025 (2020).

van Heekeren, H. R. Rock-paintings and other prehistoric discoveries near Maros (South West Celebes). *Laporan Tahunan Dinas Purbakala Republik Indonesia* **1952**, 22–35 (1952).
